# Association of inflammatory risk based on the Glasgow Prognostic Score with long-term mortality in patients with cardiovascular disease

**DOI:** 10.1038/s41598-025-90238-2

**Published:** 2025-02-22

**Authors:** Houyong Zhu, Chao Yang, Xiao Liu, Xinyu Zhu, Xiaoqun Xu, Hanxin Wang, Qilan Chen, Xiaojiang Fang, Jinyu Huang, Tielong Chen

**Affiliations:** 1 Department of Cardiology, Hangzhou TCM Hospital Affiliated to Zhejiang Chinese Medical University, No. 453 Stadium Road, Hangzhou, 310007 Zhejiang China; 2 The Fourth School of Clinical Medicine, Zhejiang Chinese Medical University, Hangzhou, Zhejiang China; 3 Zhejiang University School of Medicine, Hangzhou, Zhejiang China; 4 Hangzhou Red Cross Hospital, Hangzhou, Zhejiang China; 5 Department of Cardiology, Hangzhou First People’s Hospital, No. 261 Huansha Road, Hangzhou, 310006 Zhejiang China

**Keywords:** Cardiovascular disease, Glasgow Prognostic Score, Inflammatory risk, Mortality, Cardiology, Medical research

## Abstract

The secondary prevention strategy for cardiovascular disease (CVD) does not include anti-inflammatory treatment, which may lead to long-term inflammation in some patients. The aim of this study was to assess the association between inflammatory risk based on the Glasgow Prognostic Score (GPS) and long-term mortality risk in patients with CVD. This study included 3833 patients (≥ 20 years old) with CVD in the National Health and Nutrition Survey from 1999 to 2010 in the United States. The mortality rate was determined by correlation with the National Death Index on December 31, 2019. The GPS consists of the serum C-reactive protein and the serum albumin. The primary outcome was all-cause death, which included cardiac death and non-cardiac death. Cox proportional hazards adjusted for demographic factors and traditional cardiovascular risk factors were used to test the impact of the GPS on mortality. The sensitivity analysis was conducted on subsets within the cohort of patients with CVD, including congestive heart failure, coronary artery disease, angina, heart attack, and stroke. Among 3833 CVD patients with a median follow-up of 9.6 years, 2431 (63.4%) all-cause deaths, 822 (21.4%) cardiac deaths, and 1609 (41.9%) non-cardiac deaths were recorded. After full model adjustment, compared with those of the GPS (0) group, the hazard ratios (HRs) of all-cause death for GPS (1) and GPS (2) were 1.66 (95% confidence interval (CI), 1.48–1.86) and 2.75 (95% CI 2.01–3.75), respectively (*P* for trend < 0.001). Compared with those of the GPS (0) group, the HRs of cardiac death for the GPS (1) and GPS (2) groups were 1.69 (95% CI 1.39–2.05) and 2.18 (95% CI 1.22–3.91), respectively (*P* for trend < 0.001). Compared with those of the GPS (0) group, the HRs of non-cardiac death for the GPS (1) and GPS (2) groups were 1.65 (95% CI 1.44–1.89) and 3.05 (95% CI 2.11–4.40), respectively (*P* for trend < 0.001). The results of the sensitivity analysis were similar to those of the overall cohort. In our analysis of the United States National Database, we discovered that the GPS, a measure of inflammatory risk, was significantly associated with an increased risk of mortality among patients with CVD. Specifically, we observed that patients with a higher GPS had significantly higher risks of all-cause, cardiac, and non-cardiac mortality compared to those with a lower score. These findings suggest that the GPS, comprising easily obtainable biomarkers, could serve as a valuable tool for risk stratification in CVD patients and may contribute to the improvement of patient outcomes.

**Supplementary Information:**

The online version contains supplementary material available at 10.1038/s41598-025-90238-2.

## Introduction

Cardiovascular disease (CVD), principally ischemic heart disease (IHD) and stroke, are the leading causes of global mortality and major contributors to disability^1^. Despite the gradual increase in cardiovascular secondary prevention strategies, in 2019, 32% of the world’s deaths will still be caused by CVD, 85% of which were due to stroke and heart attack (HT) (i.e., myocardial infarction), which has seriously affected the quality of life of patients and increased the economic burden^2^. Therefore, how to effectively assess the risk of major cardiovascular adverse events is the premise of optimizing secondary prevention strategies. While traditional risk assessment models like the Framingham score are prevalent for forecasting primary cardiovascular disease (CVD) risk, the field lacks robust tools for secondary prevention^3–5^. Current forecasting models, which rely on factors including age, gender, blood pressure, glucose and lipid levels, and smoking status, face constraints and intricacies in accurately determining CVD risk. Our previous studies^6,7^ demonstrated that the Glasgow Prognostic Score (GPS), which utilizes serum albumin (ALB) and serum C-reactive protein (CRP) levels, is a straightforward and potent method for assessing the prognosis of patients experiencing acute myocardial infarction. Nonetheless, the correlation between the GPS and the onset of CVD across a broader population remains undefined. Consequently, for our investigation, we examined data across six survey cycles of the National Health and Nutrition Examination Survey (NHANES), spanning 1999 to 2010. The objective was to evaluate the influence of initial GPS on extended-term patient outcomes within the context of CVD, correlating with the National Death Index (NDI) as of December 31, 2019.

## Methods

### Ethics statement

The Institutional Review Board of the National Center for Health Statistics (NCHS) in the United States approved the NHANES, and this process also obtained informed consent from participants. Our analysis only utilized publicly accessible data from NHANES, which contained no identifiable personal information. For more detailed information, please visit https://www.cdc.gov/nchs/nhanes/about/erb.html and https://www.cdc.gov/nchs/nhanes/about/survey-content-operations.html#cdc_survey_profile_how_surveys_are_conducted-how-data-collection-works.

### National health and nutrition examination survey

NHANES is a comprehensive and stratified survey that encompasses the non-institutionalized civilian population of the United States, as administered by the NCHS. Initiated as an ongoing program in 1999, it operates on a biennial cycle. Participants in this survey undergo both a household interview and a medical examination at a portable examination center. Further details regarding the methodology of NHANES can be found in other published sources^8,9^.

**Fig. 1:**
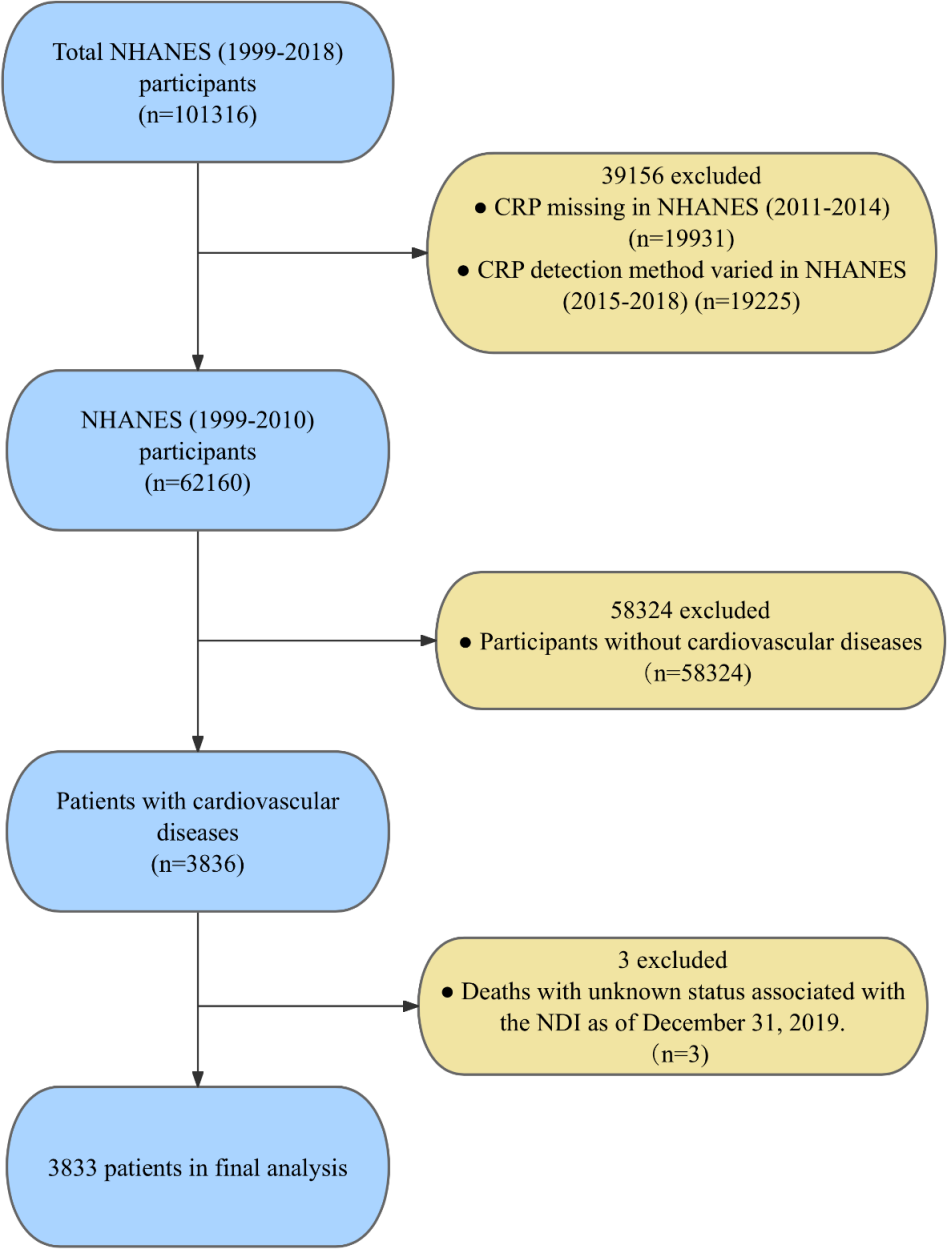
Flow diagram for recruitment of patients. CRP, C reactive protein; NDI, National Death Index; NHANES, National Health and Nutrition Examination Survey.

NHANES used latex-enhanced nephelometry for CRP detection from 1999 to 2010, and immunoturbidimetry from 2015 to 2018, which was considered to have clinical heterogeneity. CRP, as part of the GPS (main research variable), was not suitable for data consolidation. In addition, there was no detection of CRP from 2011 to 2014, so it was also excluded from the study cohort. Therefore, this data from 6 cycles of NHANES (1999–2010) were collected, and the missing CRP data or inconsistent detection methods in 4 cycles of 2011–2018 were excluded. CVD was identified by integrating self-reported diagnoses provided by physicians with responses from standardized medical questionnaires, which were part of personal interviews conducted with participants. Participants were asked the following questions: "Have doctors or other health professionals told you that you have congestive heart failure (CHF)/coronary heart disease (CHD)/angina pectoris (AP)/HT/stroke?" These five distinct questions all required a response, and any affirmative answer to any of these questions resulted in the participant being coded as CVD-positive. After excluding the non-compliant subjects, 3836 CVD positive participants were left as the subjects. The death status of these participants was determined through the association with the NDI on December 31, 2019, including all-cause death, cardiac death, and non-cardiac death^10^. After excluding 3 individuals whose death status could not be determined, the remaining 3833 CVD-positive participants were the subjects of this study (Fig. 1).

### Baseline data

Information about age, gender, race/ethnicity, education level, smoking, poverty income ratio (PIR), and disease history was collected from family interviews. Body weight and height were obtained through physical examination. Body mass index (BMI) was calculated by dividing weight by the square of height. Race/ethnicity was divided into Mexican-Americans, non-Hispanic whites, non-Hispanic blacks, and others. The education level was divided into lower than senior high school, senior high school, and higher than senior high school. PIR was was calculated by dividing the family income by the poverty threshold specific to the family size, as well as the appropriate year and state. Triglycerides, total cholesterol, and other relevant biochemical indicators were obtained from the NHANES laboratory. Hypertension was defined on the basis of a history of hypertension diagnosed by doctors, high systolic blood pressure (≥ 140 mmHg), high diastolic blood pressure (≥ 90 mmHg), or use of antihypertensive drugs. Diabetes status was defined on the basis of a history of diabetes diagnosed by doctors, high fasting blood glucose (≥ 7.0 mmol/L), high glycosylated hemoglobin (≥ 6.5%), or use of hypoglycemic drugs. Through the questionnaire survey, we also obtained the medical history of asthma, emphysema, chronic bronchitis, arthritis, cancer, liver dysfunction, etc. Liver dysfunction was defined as a history of liver disease diagnosed by a doctor or alanine aminotransferase > 3 times the upper limit of normal. The glomerular filtration rate (GFR) was calculated according to the epidemiological collaboration equation for chronic kidney disease recommended by Levey et al.^11^, and moderate or severe nephropathy was defined as a GFR < 60 mL/(min × 1.73 m^2^).

### Measurement of serum CRP and serum ALB concentrations

Participants provided blood samples via venous puncture. Samples were preserved at a designated temperature (− 20 °C) before being shipped to the University of Washington for analysis. CRP levels were measured using the latex-enhanced nephelometry, while albumin was identified through the bromcresol purple method. The precise protocols for these measurements are outlined in the NHANES Laboratory Procedure Manual^9^.

### Classification of the GPS

Individuals presenting with high CRP levels exceeding 10 mg/L and low albumin levels below 35 g/L received a score of 2 for the GPS. Those exhibiting either a CRP or albumin level abnormality were given a single point for GPS. Participants with neither CRP nor albumin levels indicating abnormality were awarded a GPS score of 0.

### Outcome events

We referenced mortality data from the NDI up to December 31, 2019. The correlation between the NHANES participants and the NDI was established by matching unique individual sequence numbers (SEQNs). The study’s primary endpoint was all-cause death, encompassing fatalities from all possible causes. Secondary endpoints included cardiac and non-cardiac mortality. The leading causes of death were categorized using the 10th revision of the international statistical classification of diseases and related health problems (ICD-10) and the standardized underlying cause of death (UCOD_LEADING) coding system developed by the NCHS. The code for cardiac death is 001, and the code for non-cardiac death includes 002–010. More information is available at https://www.cdc.gov/nchs/data/datalinkage/public-use-linked-mortality-files-data-dictionary.pdf.

### Statistical analysis

We conducted a comparison of the baseline characteristics categorized by the GPS utilizing the following methods. Given that the continuous variables exhibited a distribution that was not consistent with the normal distribution, as determined by the Kolmogorov–Smirnov test, we proceeded to calculate the median and interquartile range for these variables and subsequently employed the Kruskal–Wallis test for statistical analysis. For the categorical variables, the Pearson chi-square test was the method of choice for their classification and analysis. Considering the likelihood that covariates could influence the relationship between the GPS scores and the occurrence of outcome events, we employed the Cox proportional hazards model within a stepwise approach to calculate survival estimates. We took into account a spectrum of potential confounding factors for correction, encompassing both demographic characteristics and established heart disease risk factors. Specifically, demographic considerations included age, gender, race, educational status, smoking, PIR, and BMI. Additionally, we considered a range of cardiovascular risks such as triglyceride levels, cholesterol levels, diabetes, hypertension, asthma, emphysema, chronic bronchitis, arthritis, cancer, liver dysfunction, and moderate or severe nephropathy. Our analysis utilized a tiered approach to model adjustments: Model 1 incorporated only the fundamental demographic variables, namely age, gender, and race. Subsequently, Model 2 expanded upon Model 1 by incorporating educational status, smoking, PIR, and BMI. Finally, Model 3 was developed as our comprehensive model, which included all of the aforementioned traditional cardiovascular risk factors, augmenting the variables from Model 2. The Cox proportional hazards model, specifically Model 3, was utilized to derive the hazard ratio (HR) along with its corresponding 95% confidence interval (CI). Subsequently, a plot illustrating the cumulative risk of outcomes was constructed using the parameters from this model. Upon visual inspection of the cumulative risk standard plot and the logarithm of the negative logarithm of the Cox survival function (Fig. S1), the GPS did not have time-dependent effects. Additionally, a multicollinearity assessment was conducted, which indicated that the variance inflation factors for all variables in question were below the threshold of 10, as detailed in Table S1. These findings support the validity of the Cox model’s assumptions. For continuous variables with missing data, we employed the expectation maximization (EM) algorithm to estimate and fill in the missing values. In contrast, missing data for categorical variables were addressed by creating an additional category specifically for these unobserved values. The sensitivity analysis was completed according to specific CVD diseases, including CHF, CHD, angina pectoris, HT, and stroke.

In the secondary analysis, we conducted a subgroup analysis based on the fully adjusted model (Model 3) according to age, gender, race, smoking status, diabetes status, hypertension status, asthma status, emphysema status, chronic bronchitis, arthritis status, cancer status, liver dysfunction status, moderate or severe nephropathy, and 10-year follow-up period.

We also examined other indicators of inflammation that are pertinent to CVD prognosis, including the platelet-to-lymphocyte ratio (PLR), neutrophil-to-lymphocyte ratio (NLR), and the count of total white blood cells^12–16^. To this end, we performed a supplementary analysis post hoc to assess the predictive power of these inflammatory markers, along with CRP and ALB levels, with respect to all-cause death and other secondary endpoints. The classification of these markers into risk categories was based on the third percentile as a threshold. Consequently, patients were categorized into low risk (0) if their values fell within the first percentile, medium risk (1) for the second percentile, and high risk (3) for those within the third percentile. The HR (95% CI), number, and median (quartile) were used as summary statistics for the corresponding patients. Bilateral *P* values < 0.05 were considered to indicate statistical significance. The data were analyzed using SPSS 26.0 (SPSS, Inc., Chicago, IL).

## Results

### Patient characteristics

A total of 3833 CVD patients were ultimately included in this study (Fig. 1). The median age was 71 years, 44.4% were female, 19.2% were non-Hispanic black, and the median follow-up time was 9.6 years. The main clinical characteristics of the remaining subjects were shown in Table 1. Compared with the GPS (0) group, the GPS (≥ 1) had younger age, a higher proportion of women, a higher proportion of non-Hispanic blacks, lower PIR, higher BMI, lower total cholesterol, lower triglycerides, higher prevalence of diabetes, higher prevalence of hypertension, higher prevalence of chronic bronchitis, higher prevalence of arthritis, higher prevalence of emphysema, and higher prevalence of moderate or severe nephropathy. Missing data were present in the following variables: BMI, CRP, albumin, alanine aminotransferase, blood urea nitrogen, total cholesterol, triglyceride, creatinine, education status, PIR, smoking, hypertension, asthma, arthritis, emphysema, chronic bronchitis, liver disease, cancer, CHF, CHD, AP, HT, and stroke. The corresponding missing rates were 14.0%, 15.2%, 15.9%, 16.4%, 15.9%, 15.9%, 15.9%, 15.9%, 0.5%, 9.8%, 0.2%, 0.1%, 0.3%, 0.2%, 0.5%, 0.4%, 0.4%, 0.2%, 1.8%, 2.8%, 1.9%, 1.0%, and 0.4%, respectively. For further details, refer to Table S2.

**Table 1:** Baseline characteristics according to GPS levels in NHANES 1999–2010.

Vairable	GPS			
	0	1	2	*P* Value
Age, years	71.0 (62.0–80.0)	65.0 (56.0–76.0)	72.0 (61.0–80.0)	< 0.001
Gender (male), no. (%)	1832 (57.0)	275 (48.7)	23 (44.2)	< 0.001
Race/ethnicity, no. (%)				< 0.001
Mexican–American	437 (13.6)	77 (13.6)	3 (5.8)	
Non-Hispanic White	1983 (61.7)	297 (52.6)	25 (48.1)	
Non-Hispanic Black	570 (17.7)	145 (25.7)	21 (40.4)	
Others	226 (7.0)	46 (8.1)	3 (5.8)	
Education status, no. (%)				0.051
Less than a high school education/primary education	1285 (40.0)	252 (44.6)	26 (50.0)	
High school	780 (24.3)	135 (23.9)	6 (11.5)	
Higher than high school	1132 (35.2)	177 (31.3)	19 (36.5)	
Smoking, no. (%)	1894 (58.9)	361 (63.9)	39 (75.0)	0.038
Poverty-income ratio	2.0 (1.2–3.1)	1.8 (1.0–2.6)	1.7 (0.9–2.4)	< 0.001
Body mass index	28.4 (25.7–31.6)	31.4 (27.4–36.5)	33.5 (24.9–40.7)	< 0.001
Triglyceride, mmol/L	1.7 (1.2–2.1)	1.7 (1.2–2.3)	1.2 (0.8–2.2)	0.006
Total cholesterol, mmol/L	4.8 (4.2–5.4)	4.8 (4.2–5.6)	4.0 (3.3–5.0)	< 0.001
Diabetes, no. (%)	1057 (32.9)	233 (41.2)	27 (51.9)	< 0.001
Hypertension, no. (%)	2201 (68.4)	421 (74.5)	44 (84.6)	0.002
Asthma, no. (%)	464 (14.4)	134 (23.7)	7 (13.5)	< 0.001
Emphysema, no. (%)	233 (7.2)	69 (12.2)	5 (9.6)	0.002
Chronic bronchitis, no. (%)	374 (11.6)	88 (15.6)	13 (25.0)	0.004
Arthritis, no. (%)	1764 (54.9)	326 (57.7)	38 (73.1)	0.054
Cancer, no. (%)	666 (20.7)	105 (18.6)	16 (30.8)	0.303
Liver dysfunction, no. (%)	178 (5.5)	42 (7.4)	4 (7.7)	0.312
Moderate or severe nephropathy, no. (%)	1110 (34.5)	219 (38.8)	30 (57.7)	0.001

GPS, Glasgow Prognostic Score. Values are numbers (%) or medians (quartile).

### Primary outcome

#### All-cause death

**Fig. 2:**
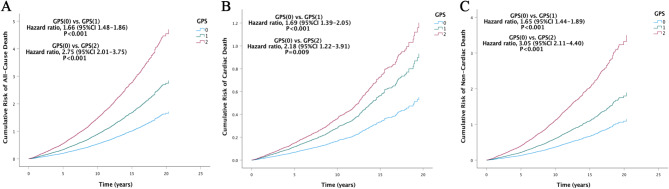
Cumulative risk of the primary and second outcomes among three GPS level groups in the model 3. (**A**) cumulative risk of all-cause death, (**B**) cumulative risk of cardiac death, (**C**) cumulative risk of non-cardiac death. GPS, Glasgow Prognostic Score.

In Model 1, compared with those in the GPS (0) group, the all-cause mortality risk in the GPS (1) [HR 1.74 (95% CI 1.56–1.94), *P* < 0.001] and GPS (2) [HR 2.99 (95% CI 2.21–4.05), *P* < 0.001] groups was greater, and the higher the GPS, the greater the all-cause mortality risk (*P* < 0.001 for trend) (Table 2 and Fig. S2). In Model 2, compared with those in the GPS (0) group, the all-cause mortality risk in the GPS (1) [HR 1.69 (95% CI 1.51–1.89), *P* < 0.001] and GPS (2) [HR 2.92 (95% CI 2.15–3.97), *P* < 0.001] groups was greater, and the higher the GPS, the greater the all-cause mortality risk (*P* < 0.001 for trend) (Table 2 and Fig. S3). According to the fully adjusted model (Model 3), compared with those in the GPS (0) group, the all-cause mortality risk associated with the GPS (1) [HR 1.66 (95% CI 1.48–1.86); *P* < 0.001] and GPS (2) [HR 2.75 (95% CI 2.01–3.75); *P* < 0.001] were greater, and the higher the GPS, the greater the all-cause mortality risk (*P* < 0.001 for trend) (Table 2 and Fig. 2). The results of the univariate model between the GPS and all-cause mortality risk were consistent with those of the three models (Table S3).

**Table 2:** Cox regression analysis for the GPS predictions of outcomes.

Outcomes	GPS, HR (95% Cl)			
	0	1	2	*P* for trend
All-cause death				
Model 1	1.00 (Reference)	1.74 (1.56–1.94)	2.99 (2.21–4.05)	< 0.001
*P* Value		< 0.001	< 0.001	
Model 2	1.00 (Reference)	1.69 (1.51–1.89)	2.92 (2.15–3.97)	< 0.001
*P* Value		< 0.001	< 0.001	
Model 3	1.00 (Reference)	1.66 (1.48–1.86)	2.75 (2.01–3.75)	< 0.001
*P* Value		< 0.001	< 0.001	
Cardiac death				
Model 1	1.00 (Reference)	1.77 (1.47–2.14)	2.45 (1.38–4.35)	< 0.001
*P* Value		< 0.001	0.002	
Model 2	1.00 (Reference)	1.72 (1.42–2.08)	2.34 (1.31–4.16)	< 0.001
*P* Value		< 0.001	0.004	
Model 3	1.00 (Reference)	1.69 (1.39–2.05)	2.18 (1.22–3.91)	< 0.001
*P* Value		< 0.001	0.009	
Non-cardiac death				
Model 1	1.00 (Reference)	1.72 (1.51–1.98)	3.27 (2.29–4.67)	< 0.001
*P* Value		< 0.001	< 0.001	
Model 2	1.00 (Reference)	1.68 (1.47–1.93)	3.23 (2.25–4.64)	< 0.001
*P* Value		< 0.001	< 0.001	
Model 3	1.00 (Reference)	1.65 (1.44–1.89)	3.05 (2.11–4.40)	< 0.001
*P* Value		< 0.001	< 0.001	

Model 1 is adjusted for age, sex, and race. Model 2 is adjusted for variables in Model 1 + education status, smoking, poverty-income ratio, and body mass index. Model 3 is adjusted for variables in Model 2 + triglyceride, total cholesterol, diabetes, hypertension, asthma, emphysema, chronic bronchitis, arthritis, cancer, liver dysfunction, and moderate or severe nephropathy. CI: confidence interval, GPS: Glasgow Prognostic Score, HR: hazard ratio.

### Secondary outcome

#### Cardiac death

In Model 1, compared with those in the GPS (0) group, the cardiac mortality risk in the GPS (1) [HR 1.77 (95% CI 1.47–2.14), *P* < 0.001] and GPS (2) [HR 2.45 (95% CI 1.38–4.35), *P* = 0.002] groups were greater, and the higher the GPS, the greater the cardiac mortality risk (*P* < 0.001 for trend) (Table 2 and Fig. S2). In Model 2, compared with those in the GPS (0) group, the cardiac mortality risk in the GPS (1) [HR 1.72 (95% CI 1.42–2.08), *P* < 0.001] and GPS (2) [HR 2.34 (95% CI 1.31–4.16), *P* = 0.004] groups were greater, and the higher the GPS, the greater the increase in cardiac mortality risk (*P* < 0.001 for trend) (Table 2 and Fig. S3). According to the fully adjusted model (Model 3), compared with those in the GPS (0) group, the cardiac mortality risk in the GPS (1) [HR 1.69 (95% CI 1.39–2.05), *P* < 0.001] and GPS (2) [HR 2.18 (95% CI 1.22–3.91), *P* = 0.009] groups were greater, and the higher the GPS, the greater the cardiac mortality risk (*P* < 0.001 for trend) (Table 2 and Fig. 2). The results of the univariate model between the GPS and cardiac mortality risk were consistent with those of the three models (Table S3).

### Non-cardiac death

In Model 1, compared with those in the GPS (0) group, the non-cardiac mortality risk in the GPS (1) [HR 1.72 (95% CI 1.51–1.98), *P* < 0.001] and GPS (2) [HR 3.27 (95% CI 2.29–4.67), *P* < 0.001] groups was greater. The higher the GPS, the greater the non-cardiac mortality risk was (Table 2 and Fig. S2). In Model 2, compared with those in the GPS (0) group, the non-cardiac mortality risk in the GPS (1) [HR 1.68 (95% CI 1.47–1.93), *P* < 0.001] and GPS (2) [HR 3.23 (95% CI 2.25–4.64), *P* < 0.001] groups were greater. The higher the GPS, the greater the non-cardiac mortality risk was (*P* < 0.001 for trend) (Table 2 and Fig. S3). In the fully adjusted model (Model 3), compared with the GPS (0) group, the non-cardiac mortality risk of GPS (1) [HR 1.65 (95% CI 1.44–1.89), *P* < 0.001] and GPS (2) [HR 3.05 (95% CI 2.11–4.40), *P* < 0.001] was higher. The higher the GPS, the greater the non-cardiac mortality risk was (*P* < 0.001 for trend) (Table 2 and Fig. 2). The results of the univariate model between the GPS and non-cardiac mortality risk were consistent with those of the three models (Table S3).

### Sensitivity analysis

In the CHF cohort, compared with those in the GPS (0) group, the risks of all-cause mortality, cardiac mortality, and non-cardiac mortality associated with the GPS (1) and GPS (2) increased. The higher the GPS, the greater the risks of all outcomes were (all *P* < 0.001 for trend) (Table 3). In the CHD cohort, compared with those in the GPS (0) group, the risks of all-cause mortality, cardiac mortality, and non-cardiac mortality were greater for the GPS (1) and GPS (2) groups. The higher the GPS, the greater the risks of all outcomes were (all *P* < 0.001 for trend). In the angina cohort, compared with those in the GPS (0) group, the risks of all-cause mortality and non-cardiac mortality associated with the GPS (1) and GPS (2) increased. The higher the GPS, the greater the risks of all-cause mortality and non-cardiac mortality were (both *P* < 0.001 for trend); however, there was no significant difference between the GPS and cardiac mortality risk (*P* = 0.358 for trend). In the HT cohort, compared with those in the GPS (0) group, the risks of all-cause mortality, cardiac mortality, and no-cardiac mortality associated with the GPS (1) and GPS (2) increased. The higher the GPS, the greater the risks of all outcomes were (all *P* < 0.001 for trend). In the stroke cohort, compared with those in the GPS (0) group, the risks of all-cause mortality, cardiac mortality, and non-cardiac mortality associated with the GPS (1) and GPS (2) increased. The greater the GPS, the greater the risks of all outcomes were (all *P* < 0.05 for trend). According to the sensitivity analysis, the effect of the GPS on all-cause mortality risk was consistent with that of the total cohort (Fig. S4).

**Table 3:** Sensitivity analyses for the individual CVD components.

Outcomes based on Model 3	CHF, HR (95% Cl)	*P* Value	CHD, HR (95% Cl)	*P* Value	Angina, HR (95% Cl)	*P* Value	HT, HR (95% Cl)	*P* Value	Stroke, HR (95% Cl)	*P* Value
All-cause death										
GPS										
0	1.00 (Reference)		1.00 (Reference)		1.00 (Reference)		1.00 (Reference)		1.00 (Reference)	
1	1.72 (1.43–2.07)	< 0.001	1.77 (1.46–2.15)	< 0.001	1.58 (1.26–1.98)	< 0.001	1.57 (1.32–1.87)	< 0.001	1.55 (1.28–1.87)	< 0.001
2	2.25 (1.48–3.41)	< 0.001	3.32 (1.85–5.95)	< 0.001	2.78 (1.50–5.16)	0.001	2.21 (1.27–3.84)	0.005	4.03 (2.45–6.60)	< 0.001
*P* for trend	< 0.001		< 0.001		< 0.001		< 0.001		< 0.001	
Cardiac death										
GPS										
0	1.00 (Reference)		1.00 (Reference)		1.00 (Reference)		1.00 (Reference)		1.00 (Reference)	
1	2.16 (1.63–2.88)	< 0.001	1.83 (1.32–2.54)	< 0.001	1.16 (0.76–1.77)	0.485	1.63 (1.22–2.17)	< 0.001	1.64 (1.19–2.27)	0.003
2	2.66 (1.41–5.04)	0.003	5.11 (2.22–11.79)	< 0.001	1.49 (0.46–4.82)	0.503	2.95 (1.34–6.47)	0.007	1.53 (0.38–6.27)	0.552
*P* for trend	< 0.001		< 0.001		0.358		< 0.001		0.004	
Non-cardiac death										
GPS										
0	1.00 (Reference)		1.00 (Reference)		1.00 (Reference)		1.00 (Reference)		1.00 (Reference)	
1	1.49 (1.16–1.90)	0.002	1.75 (1.37–2.22)	< 0.001	1.84 (1.40–2.42)	< 0.001	1.54 (1.24–1.92)	< 0.001	1.51 (1.20–1.90)	< 0.001
2	1.95 (1.12–3.39)	0.018	2.41 (1.06–5.49)	0.037	3.79 (1.83–7.85)	< 0.001	1.72 (0.79–3.73)	0.169	5.16 (3.03–8.79)	< 0.001
*P* for trend	< 0.001		< 0.001		< 0.001		< 0.001		< 0.001	

Model 3 is adjusted for variables in age, gender, race, education status, smoking, poverty-income ratio, body mass index, triglyceride, total cholesterol, diabetes, hypertension, asthma, emphysema, chronic bronchitis, arthritis, cancer, liver dysfunction, and moderate or severe nephropathy. CHD: Coronary heart disease, CHF: congestive heart failure, CI: confidence interval, CVD, cardiovascular diseases, GPS: Glasgow Prognostic Score, HR: hazard ratio, HT: Heart attack.

### Subgroup analysis

In the subgroups determined according to age, gender, race, smoking status, diabetes status, hypertension status, asthma status, emphysema status, chronic bronchitis status, arthritis status, cancer status, liver dysfunction status, moderate or severe nephropathy status, and 10-year follow-up period, the impact of GPS on the risks of primary and secondary outcomes was similar (Table S4). Further interaction tests revealed that the effect of the GPS on all-cause mortality risk differed according to age, arthritis status, liver dysfunction status, and follow-up time. The effect of GPS on cardiac mortality risk differed according to asthma, chronic bronchitis, arthritis, liver dysfunction, and follow-up time. The effect of the GPS on non-cardiac mortality risk differed according to age and arthritis status.

### Post hoc analysis

According to the fully adjusted model (Model 3), the association between PLR and the risk of all-cause death was not statistically significant (*P* for trend = 0.366). However, the higher the NLR, total white blood cell count, CRP level, and albumin level, the greater the risk of all-cause death was (all *P* < 0.001 for trend). The PLR was not significantly associated with the risk of cardiac death (*P* = 0.262 for trend). However, the higher the NLR, total white blood cell count, CRP level, and albumin level, the greater the risk of cardiac death was (*P* < 0.001, *P* = 0.005, *P* < 0.001, *P* < 0.001 for trend, respectively). The PLR was not significantly associated with the risk of non-cardiac deS5ath (*P* for trend = 0.739). However, the higher the NLR, total white blood cell count, CRP level, and albumin level, the greater the risk of non-cardiac death was (all *P* < 0.001 for trend) (Table ).

## Discussion

In our analysis of data from the United States NHANES, we discovered that baseline inflammation risk, as assessed by the GPS, was significantly associated with increased risks of all-cause mortality, cardiac mortality, and non-cardiac mortality, after adjusting for potential confounders. Furthermore, we observed that a higher GPS corresponded to a greater risk of all-cause, cardiac, and non-cardiac death. The sensitivity analysis, which included conditions such as CHF, CHD, angina, HT, and stroke, confirmed the consistency of these findings with the overall cohort. To our knowledge, this study is the first to leverage national data to explore the long-term prognostic significance of the GPS in CVD patients.

GPS incorporates levels of CRP and ALB. CRP is recognized as a significant inflammatory biomarker^17,18^, with numerous studies affirming its strong predictive capabilities for CVD^19–21^. On the other hand, serum ALB levels are traditionally viewed as a nutritional status indicator. However, it has been discovered that ALB possesses multiple binding sites that serve as an effective scaffold for neutralizing free radicals. This property endows ALB with robust anti-inflammatory and antioxidant properties. Furthermore, ALB interacts with various inflammatory mediators, playing a crucial role in modulating the immune system’s response to systemic inflammation^22,23^; therefore, the GPS may succinctly and partly reflect the level of inflammation and immunity in patients. Our previous studies^6,7^ suggested that the GPS could independently predict the risk of major adverse cardiovascular events during hospitalization in patients with myocardial infarction. The strengths of this study include compensating for the lack of follow-up data from previous studies and expanding the subject population and sample size. In a broad CVD cohort with a median follow-up of 9.6 years, the baseline GPS was associated with death, and a higher GPS was associated with increased mortality, suggesting that the initially assessed GPS has an important impact on long-term outcomes in CVD patients, which may be very important.

The importance of inflammatory and immunological doctrine in the initiation and progression of atherosclerosis has received renewed attention. Adamstein et al.^12^ combined data from five randomized controlled trials (RCTs) and revealed that leukocyte levels independently predict cardiovascular events and death. The secondary prevention strategies for atherosclerotic CVD include lipid-lowering agents, antithrombotic agents, blood pressure lowering agents, and cardiovascular remodeling improvement; however, these strategies do not include anti-inflammatory or immune therapy, which may result in a long-term hyperinflammatory and immune dysregulation state in some CVD patients. Despite the current guidelines recommending intensive lipid-lowering therapy^24–26^, however, numerous clinical trials of statins, nonstatin agents, and combination therapies demonstrated a sustained residual risk of CVD despite aggressive low-density lipoprotein cholesterol (LDL-C) lowering^27–29^, which may be related to ongoing inflammation after intensive LDL-C therapy^30^. Three RCTs, COLCOT [Colchicine Cardiovascular Outcomes Trial]^31^, CANTOS [Canakinumab Antiinflammatory Thrombosis Outcome Study]^32^, and CIRT [Cardiovascular Inflammation Reduction Trial]^33^, were designed to evaluate the prognosis of patients with myocardial infarction receiving anti-inflammatory treatment. Patients in the first two studies had higher baseline CRP levels, and their results showed the positive value of anti-inflammatory treatment, while the baseline CRP level of patients included in the CIRT was only 1.6 mg/L. The results showed that anti-inflammatory treatment did not improve the prognosis of patients, which may partly explain why the initial assessment of the GPS still has a profound impact on the prognosis of patients with CVD.

The OPTIcal-COherence Tomography in Acute Coronary Syndrome (OPTIMO-ACS) study^34^ analyzed the local immune response of the criminal plaque microenvironment in patients with acute coronary syndrome (ACS). The number of CD8 + T lymphocytes detected in thrombi extracted from the blood vessels of patients with complete fibrous cap ACS was significantly increased. Broch et al.^35^ conducted a study of tocilizumab (a recombinant humanized anti-human interleukin 6 receptor monoclonal antibody) versus a placebo in patients with ST-segment elevation myocardial infarction and showed that patients receiving tocilizumab had a significantly greater myocardial salvage index. Kyaw et al.^36^ used an anti-CD20 antibody to deplete B cells from myocardial infarction model mice and found that the acceleration of atherosclerosis induced by myocardial infarction was decreased. These results suggest that long-term high inflammation or abnormal immune status in the body increases the risk of adverse cardiovascular events, and effective evaluation of inflammation and immune levels, as well as active intervention, may be important for ensuring the prognosis of patients. It was noteworthy that the association between GPS and non-cardiac mortality risk was not weaker than its association with cardiac mortality risk, indicating that GPS assessment of inflammation was not limited to the cardiovascular system. In fact, the GPS was first proposed by Forrest et al.^37^ and was found to predict the survival of non-small cell lung cancer patients, and subsequent studies^38–40^ confirmed its ability to predict the prognosis of patients with various types of cancer. Moreover, recent studies showed that the ratio based on CRP and albumin has predictive value for the prognosis of pancreatitis^41^, sepsis^42^, COVID-19^43,44^, pulmonary embolism^45^, and even Parkinson’s disease patients^46^. These findings, in concordance with our results, highlight the GPS’s potential as a pivotal tool for assessing inflammation across a broad spectrum of diseases.

There were also some interesting findings in the subgroup analysis. First, in patients with asthma and chronic bronchitis, the GPS did not reflect the risk of cardiac death. In patients with arthritis, although the GPS reflected the risk of cardiac death, the correlation was still lower than that in non-arthritic patients, which indicated that inflammation and immune diseases outside the cardiovascular system may reduce the correlation between the GPS and the risk of cardiac death. Second, the GPS was more valuable for assessing the risk of death in patients aged 65 years and older than in those younger than 65 years. Furthermore, the GPS was more valuable for assessing the risk of death in patients with less than or equal to 10 years of follow-up than in patients with more than 10 years of follow-up. Post hoc analysis revealed that the GPS, NLR, and total white blood cell count had a better predictive value for long-term mortality risk than the PLR. CRP and ALB alone also possessed good predictive value for long-term mortality risk, which was consistent with the results of the GPS. However, the definitions of NLR and total white blood cell count have varied significantly across numerous prior studies, with each employing different cutoff values^12,14,15^. In contrast, the GPS provides a singular, clear definition that may offer greater clinical utility and consistency.

This study had several limitations. First, this broad cohort of CVD was collected by the staff of NHANES. We respected the authenticity of the data collected by these professionals, yet it should be noted that the composition of this cohort did not entirely align with previous cohorts of CVD. However, the results of our sensitivity analysis were in close agreement with the overall findings, thus making it highly unlikely that our methods would have a material impact on the conclusions drawn. Second, the inability to equally allocate participants to each group and the presence of missing baseline data are inherent limitations of retrospective studies. In addition, the purpose of this study was to explore the impact of baseline GPS on the mortality risk of CVD patients. Dynamic monitoring of the GPS scores has not been completed, so it is impossible to assess the impact of dynamic changes in the GPS scores on the outcomes of CVD patients.

## Conclusions

The GPS, serving as an indicator of inflammation risk, is closely associated with the long-term mortality risk in patients with CVD. A higher GPS is indicative of a greater risk of death. Therefore, the GPS may serve as a convenient and efficient practical clinical risk assessment tool for CVD patients. However, large-scale and prospective clinical trials are still necessary to evaluate the effectiveness of the GPS and to further assess whether improvements in GPS scores significantly impact the outcomes for CVD patients.

## Electronic supplementary material

Below is the link to the electronic supplementary material.

Supplementary Material 1

## Data Availability

Publicly available datasets were analyzed in this study. This data can be found here: https://www.cdc.gov/nchs/nhanes.

## Declarations

### Patient and public involvement

Patients and/or the public were not involved in the design, or conduct, or reporting, or dissemination plans of this research.
