# Supplementary material for: Association of inflammatory risk based on the Glasgow Prognostic Score with long-term mortality in patients with cardiovascular disease
Source: Sci Rep. 2025 Feb 22;15:6474. doi: 10.1038/s41598-025-90238-2 (PMC11846972; doi:10.1038/s41598-025-90238-2)
Supplement: Supplementary file 1 — Supplementary Material 1 [file 41598_2025_90238_MOESM1_ESM.docx]

**Supplementary Online Content**

Association of inflammatory risk based on the Glasgow Prognostic Score with long-term mortality in patients with cardiovascular disease

**Catalogue**

[S1 Fig. Evaluation of non time-dependent effects of Cox survival function 3](#_Toc804312286)

[S1 Table. Multicollinearity test for all-cause death 4](#_Toc1814516584)

[S2 Table. Statistics of missing value and extremesa 5](#_Toc164956241)

[S2 Fig. Cumulative incidence of the primary and second outcomes among three GPS level groups in the model 1 6](#_Toc18154210)

[S3 Fig. Cumulative incidence of the primary and second outcomes among three GPS level groups in the model 2 7](#_Toc175129596)

[S3 Table. Cox regression analysis for the GPS predictions of outcomes 8](#_Toc1350523582)

[S4 Table. Subgroups analyses of the effect of GPS level on adverse events based on Model 3 9](#_Toc1495177531)

[S5 Table. Cox regression analysis for the post hoc analysis outcomes 11](#_Toc1742609970)

[S4 Fig. Consistent Association: GPS with Mortality Risk in Cardiovascular Disease Patients 12](#_Toc663788004)

## S1 Fig. Evaluation of non time-dependent effects of Cox survival function


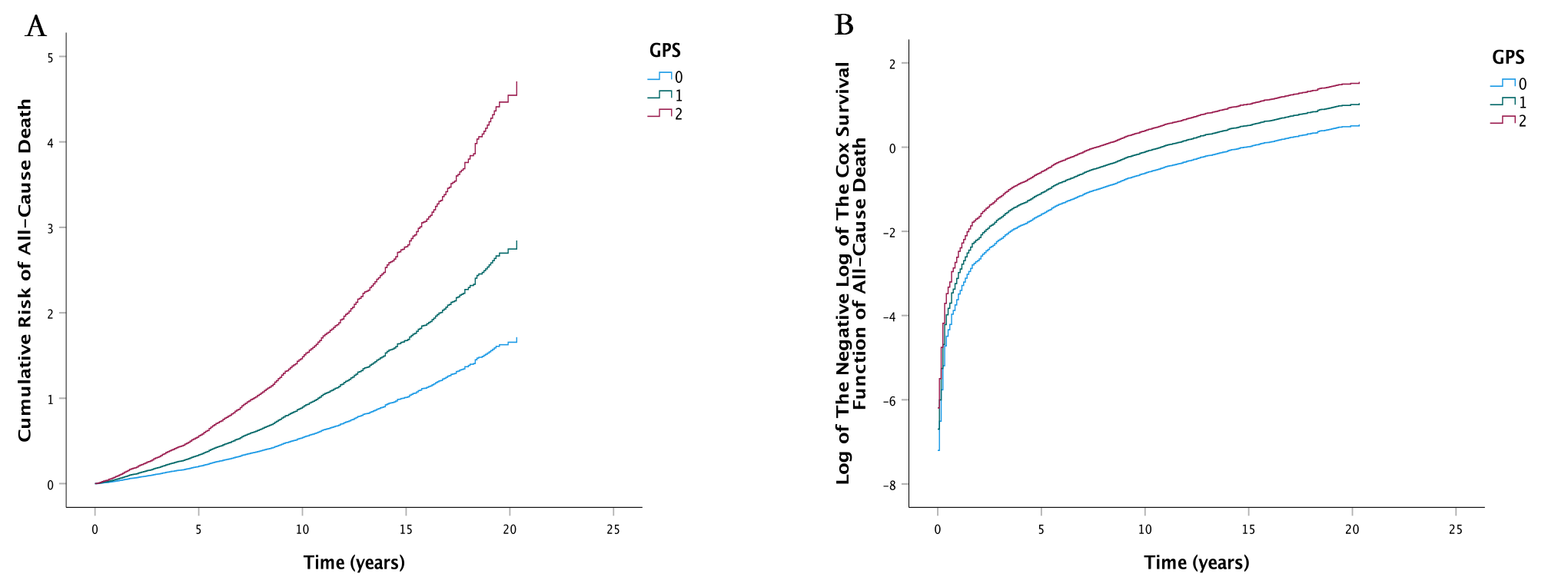


S1 Fig. Evaluation of non time-dependent effects of Cox survival function in the model 3. A. cumulative risk standard plot of all-cause death, B. logarithm of the negative logarithm of the Cox survival function of all-cause death. GPS: Glasgow Prognostic Score.

## S1 Table. Multicollinearity test for all-cause death

| **S1 Table. Multicollinearity test for all-cause death** | | | | | | | |
| --- | --- | --- | --- | --- | --- | --- | --- |
| Model 3 | Unstandardized Coefficients | | Standardized Coefficients | t | Sig. | Collinearity Statistics | |
|  | B | Std. Error | Beta |  |  | Tolerance | VIF |
| (Constant) | -0.449 | 0.072 |  | -6.243 | 0.000 |  |  |
| Age | 0.016 | 0.001 | 0.440 | 28.142 | 0.000 | 0.733 | 1.365 |
| Gender | 0.043 | 0.014 | 0.045 | 3.030 | 0.002 | 0.831 | 1.203 |
| Race | -0.014 | 0.009 | -0.023 | -1.638 | 0.102 | 0.946 | 1.057 |
| Education status | -0.017 | 0.008 | -0.031 | -2.224 | 0.026 | 0.933 | 1.072 |
| Smoking | 0.069 | 0.014 | 0.070 | 4.928 | 0.000 | 0.881 | 1.135 |
| PIR | -0.036 | 0.007 | -0.075 | -5.407 | 0.000 | 0.941 | 1.063 |
| Body mass index | -0.004 | 0.001 | -0.046 | -3.115 | 0.002 | 0.808 | 1.237 |
| Triglyceride | -0.008 | 0.005 | -0.025 | -1.646 | 0.100 | 0.803 | 1.246 |
| Total cholesterol | 0.018 | 0.006 | 0.041 | 2.738 | 0.006 | 0.791 | 1.264 |
| Diabetes | 0.061 | 0.014 | 0.060 | 4.235 | 0.000 | 0.888 | 1.126 |
| Hypertension | -0.010 | 0.014 | -0.010 | -0.709 | 0.478 | 0.930 | 1.075 |
| Asthma | -0.009 | 0.019 | -0.007 | -0.477 | 0.634 | 0.835 | 1.198 |
| Emphysema | 0.113 | 0.023 | 0.071 | 4.962 | 0.000 | 0.868 | 1.152 |
| Chronic bronchitis | 0.001 | 0.020 | 0.001 | 0.036 | 0.971 | 0.852 | 1.174 |
| Arthritis | -0.032 | 0.014 | -0.033 | -2.333 | 0.020 | 0.912 | 1.096 |
| Cancer | -0.008 | 0.016 | -0.007 | -0.503 | 0.615 | 0.945 | 1.058 |
| Liver dysfunction | 0.028 | 0.024 | 0.015 | 1.141 | 0.254 | 0.984 | 1.016 |
| Moderate or severe nephropathy | 0.188 | 0.015 | 0.186 | 12.689 | 0.000 | 0.832 | 1.202 |
| GPS | 0.114 | 0.016 | 0.098 | 7.072 | 0.000 | 0.928 | 1.078 |

S1 Table. Multicollinearity test for all-cause death. BMI: body mass index, GPS: Glasgow Prognostic Score, no.: number, PIR: poverty-income ratio, Tch: total cholesterol, TG: triglyceride, VIF: variance inflation factor.

## S2 Table. Statistics of missing value and extremesa

|  | **N** | **Mean** | **Std. Deviation** | **Missing** | | **No. of Extremes^a^** | |
| --- | --- | --- | --- | --- | --- | --- | --- |
|  |  |  |  | Count | Percent | Low | High |
| Age, years | 3833 | 68.26 | 13.193 | 0 | .0 | 60 | 0 |
| Sex, no. | 3833 |  |  | 0 | .0 |  |  |
| BMI, kg/m^2^ | 3298 | 29.72 | 6.746 | 535 | 14.0 | 0 | 98 |
| CRP, mg/L | 3252 | 6.54 | 12.642 | 581 | 15.2 | 0 | 308 |
| Albumin, g/L | 3224 | 41.37 | 3.532 | 609 | 15.9 | 31 | 3 |
| ALT, U/L | 3205 | 23.46 | 19.482 | 628 | 16.4 | 0 | 214 |
| BUN, mmol/L | 3223 | 6.41 | 3.466 | 610 | 15.9 | 0 | 184 |
| Tch, mmol/L | 3224 | 4.92 | 1.218 | 609 | 15.9 | 0 | 51 |
| TG, mmol/L | 3222 | 1.90 | 1.611 | 611 | 15.9 | 0 | 163 |
| Creatinine, μmol/L | 3223 | 100.40 | 75.371 | 610 | 15.9 | 0 | 208 |
| Education status, no. | 3812 |  |  | 21 | .5 |  |  |
| PIR | 3459 | 2.26 | 1.49 | 374 | 9.8 |  |  |
| Race/ethnicity, no. | 3833 |  |  | 0 | .0 |  |  |
| Smoking, no. | 3827 |  |  | 6 | .2 |  |  |
| Hypertension, no. | 3827 |  |  | 6 | .1 |  |  |
| Diabetes, no. | 3833 |  |  | 0 | .0 |  |  |
| Asthma, no. | 3823 |  |  | 10 | .3 |  |  |
| Arthritis, no. | 3826 |  |  | 7 | .2 |  |  |
| Emphysema, no. | 3813 |  |  | 20 | .5 |  |  |
| Chronic bronchitis, no. | 3818 |  |  | 15 | .4 |  |  |
| Liver disease, no. | 3816 |  |  | 17 | .4 |  |  |
| Cancer, no. | 3825 |  |  | 8 | .2 |  |  |
| CHF, no. | 3764 |  |  | 69 | 1.8 |  |  |
| CHD, no. | 3727 |  |  | 106 | 2.8 |  |  |
| AP, no. | 3759 |  |  | 74 | 1.9 |  |  |
| HT, no. | 3796 |  |  | 37 | 1.0 |  |  |
| Stroke, no. | 3819 |  |  | 14 | .4 |  |  |
| Follow up duration, months | 3833 | 111.09 | 63.654 | 0 | .0 | 0 | 0 |
| a Number of cases outside the range (Q1 - 1.5*IQR, Q3 + 1.5*IQR). | | | | | | | |

S2 Table. Statistics of missing value and extremesa. AP: angina pectoris, ALT: alanine aminotransferase, BMI: body mass index, BUN: blood urea nitrogen, CHD: coronary heart disease, CHF: congestive heart failure, CRP: C reactive protein, HT: heart attack, no.: number, PIR: poverty-income ratio, Tch: total cholesterol, TG: triglyceride.

## S2 Fig. Cumulative incidence of the primary and second outcomes among three GPS level groups in the model 1


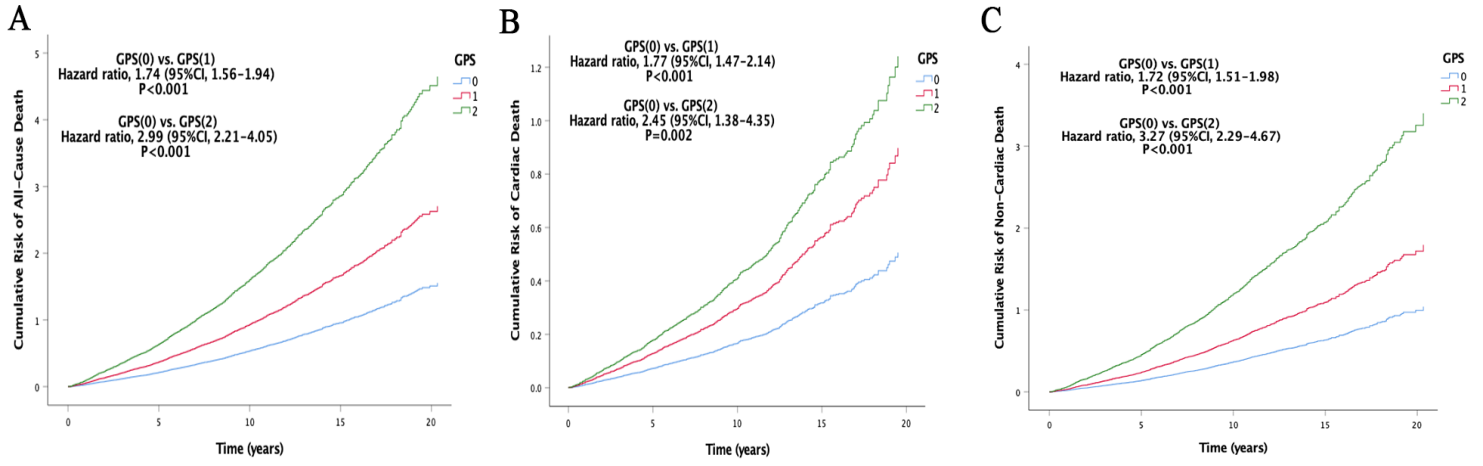


S2 Fig. Cumulative incidence of the primary and second outcomes among three GPS level groups in the model 1. A. cumulative risk of all-cause death, B. cumulative risk of cardiac death, C. cumulative risk of non-cardiac death. GPS: Glasgow Prognostic Score.

## S3 Fig. Cumulative incidence of the primary and second outcomes among three GPS level groups in the model 2


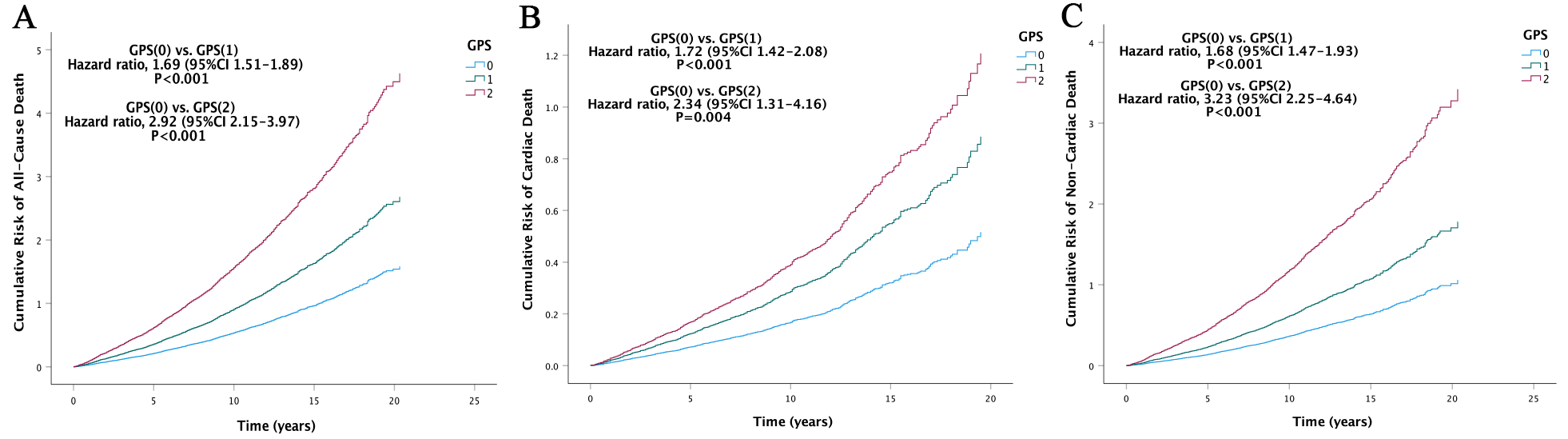


S3 Fig. Cumulative incidence of the primary and second outcomes among three GPS level groups in the model 2. A. cumulative risk of all-cause death, B. cumulative risk of cardiac death, C. cumulative risk of non-cardiac death. GPS: Glasgow Prognostic Score.

## S3 Table. Cox regression analysis for the GPS predictions of outcomes

| **S3 Table. Cox regression analysis for the GPS predictions of outcomes** | | | | |
| --- | --- | --- | --- | --- |
| Outcomes | GPS, HR (95% Cl) | | | |
|  | 0 | 1 | 2 | P for trend |
| All-cause death |  |  |  |  |
| Model 0 | 1.00 (Reference) | 1.28 (1.15-1.43) | 2.32 (1.71-3.14) | <0.001 |
| P Value |  | <0.001 | <0.001 |  |
| Cardiac death |  |  |  |  |
| Model 0 | 1.00 (Reference) | 1.27 (1.05-1.53) | 1.89 (1.07-3.35) | 0.002 |
| P Value |  | 0.012 | 0.029 |  |
| Non-cardiac death |  |  |  |  |
| Model 0 | 1.00 (Reference) | 1.29 (1.13-1.47) | 2.54 (1.78-3.63) | <0.001 |
| P Value |  | <0.001 | <0.001 |  |

S3 Table. Cox regression analysis for the GPS predictions of outcomes. Model 0 is the result between GPS and mortality risk without adjustment. CI: confidence interval, GPS: Glasgow Prognostic Score.

## S4 Table. Subgroups analyses of the effect of GPS level on adverse events based on Model 3

| **S4 Table. Subgroups analyses of the effect of GPS level on adverse events based on Model 3** | | | | | | | | | | | | | | | |
| --- | --- | --- | --- | --- | --- | --- | --- | --- | --- | --- | --- | --- | --- | --- | --- |
|  | Primary and secondary outcomes | | | | | | | | | | | | | | |
| Subgroup | All-cause death | | | P for trend | P for interaction | Cardiac death | | | P for trend | P for interaction | Non-cardiac death | | | P for trend | P for interaction |
|  | GPS, HR (95% Cl) | | |  |  | GPS, HR (95% Cl) | | |  |  | GPS, HR (95% Cl) | | |  |  |
| Age | 0 | 1 | 2 |  |  | 0 | 1 | 2 |  |  | 0 | 1 | 2 |  |  |
| ≥65 year | 1.00 (Reference) | 1.42 (1.24-1.62) | 2.52 (1.76-3.61) | <0.001 | 0.001 | 1.00 (Reference) | 1.50 (1.20-1.87) | 1.84 (0.94-3.61) | <0.001 | 0.275 | 1.00 (Reference) | 1.38 (1.17-1.63) | 2.93 (1.91-4.49) | <0.001 | 0.002 |
| ＜65 year | 1.00 (Reference) | 1.86 (1.50-2.31) | 2.13 (1.13-4.04) | <0.001 |  | 1.00 (Reference) | 1.55 (1.04-2.29) | 2.05 (0.61-6.87) | 0.019 |  | 1.00 (Reference) | 2.03 (1.56-2.63) | 2.17 (1.02-4.61) | <0.001 |  |
| Gender |  | | | | |  | | | | |  | | | | |
| Male | 1.00 (Reference) | 1.74 (1.50-2.03) | 3.28 (2.12-5.10) | <0.001 | 0.090 | 1.00 (Reference) | 1.76 (1.36-2.29) | 3.06 (1.43-6.56) | <0.001 | 0.209 | 1.00 (Reference) | 1.74 (1.44-2.10) | 3.40 (1.98-5.83) | <0.001 | 0.228 |
| Female | 1.00 (Reference) | 1.61 (1.37-1.91) | 2.45 (1.57-3.82) | <0.001 |  | 1.00 (Reference) | 1.65 (1.24-2.21) | 1.60 (0.64-3.98) | 0.001 |  | 1.00 (Reference) | 1.60 (1.31-1.96) | 2.89 (1.74-4.80) | <0.001 |  |
| Race |  | | | | |  | | | | |  | | | | |
| Mexican-American | 1.00 (Reference) | 2.12 (1.53-2.92) | 6.14 (1.86-20.28) | <0.001 | 0.854 | 1.00 (Reference) | 2.08 (1.13-3.83) | 6.55 (0.82-52.58) | 0.006 | 0.839 | 1.00 (Reference) | 2.14 (1.47-3.13) | 5.99 (1.39-25.80) | <0.001 | 0.706 |
| Non-Hispanic White | 1.00 (Reference) | 1.65 (1.43-1.91) | 2.11 (1.36-3.27) | <0.001 |  | 1.00 (Reference) | 1.68 (1.31-2.15) | 1.80 (0.84-3.88) | <0.001 |  | 1.00 (Reference) | 1.63 (1.36-1.96) | 2.29 (1.33-3.92) | <0.001 |  |
| Non-Hispanic Black | 1.00 (Reference) | 1.45 (1.13-1.88) | 3.31 (1.92-5.72) | <0.001 |  | 1.00 (Reference) | 1.61 (1.05-2.46) | 2.28 (0.68-7.58) | 0.015 |  | 1.00 (Reference) | 1.37 (0.99-1.89) | 3.66 (1.96-6.83) | <0.001 |  |
| Others | 1.00 (Reference) | 2.46 (1.51-3.99) | 20.57 (5.33-79.29) | <0.001 |  | 1.00 (Reference) | 1.92 (0.74-4.97) | - | 0.042 |  | 1.00 (Reference) | 2.79 (1.57-4.95) | 28.35 (4.78-168.04) | <0.001 |  |
| Smoking |  | | | | |  | | | | |  | | | | |
| Yes | 1.00 (Reference) | 1.59 (1.38-1.82) | 2.72 (1.89-3.92) | <0.001 | 0.634 | 1.00 (Reference) | 1.68 (1.32-2.14) | 1.99 (0.97-4.10） | <0.001 | 0.484 | 1.00 (Reference) | 1.54 (1.30-1.83) | 3.10 (2.03-4.73) | <0.001 | 0.924 |
| No | 1.00 (Reference) | 1.78 (1.46-2.16) | 2.46 (1.32-4.56) | <0.001 |  | 1.00 (Reference) | 1.72 (1.24-2.40) | 2.63 (0.95-7.29) | <0.001 |  | 1.00 (Reference) | 1.81 (1.42-2.30) | 2.32 (1.07-5.04) | <0.001 |  |
| Diabetes |  | | | | |  | | | | |  | | | | |
| Yes | 1.00 (Reference) | 1.73 (1.44-2.07) | 2.96 (1.91-4.60) | <0.001 | 0.451 | 1.00 (Reference) | 1.66 (1.21-2.27) | 2.80 (1.25-6.28) | <0.001 | 0.801 | 1.00 (Reference) | 1.76 (1.42-2.20) | 3.03 (1.79-5.12) | <0.001 | 0.452 |
| No | 1.00 (Reference) | 1.59 (1.37-1.84) | 2.54 (1.61-4.02) | <0.001 |  | 1.00 (Reference) | 1.64 (1.27-2.10) | 1.77 (0.73-4.31) | <0.001 |  | 1.00 (Reference) | 1.56 (1.31-1.87) | 2.95 (1.73-5.04) | <0.001 |  |
| Hypertension |  | | | | |  | | | | |  | | | | |
| Yes | 1.00 (Reference) | 1.68 (1.47-1.92) | 2.75 (1.97-3.85) | <0.001 | 0.932 | 1.00 (Reference) | 1.58 (1.25-1.99) | 2.04 (1.07-3.88) | <0.001 | 0.147 | 1.00 (Reference) | 1.73 (1.47-2.04) | 3.15 (2.12-4.67) | <0.001 | 0.270 |
| No | 1.00 (Reference) | 1.64 (1.32-2.04) | 2.71 (1.09-6.70) | <0.001 |  | 1.00 (Reference) | 2.05 (1.42-2.97) | 4.12 (0.98-17.38) | <0.001 |  | 1.00 (Reference) | 1.48 (1.13-1.94) | 2.15 (0.67-6.89) | 0.002 |  |
| Asthma |  | | | | |  | | | | |  | | | | |
| Yes | 1.00 (Reference) | 1.48 (1.13-1.95) | 2.43 (0.96-6.19) | 0.002 | 0.470 | 1.00 (Reference) | 1.08 (0.64-1.84) | - | 0.976 | 0.019 | 1.00 (Reference) | 1.68 (1.22-2.32) | 3.89 (1.49-10.18) | <0.001 | 0.456 |
| No | 1.00 (Reference) | 1.72 (1.52-1.94) | 2.87 (2.06-3.99) | <0.001 |  | 1.00 (Reference) | 1.88 (1.52-2.31) | 2.58 (1.43-4.64) | <0.001 |  | 1.00 (Reference) | 1.64 (1.40-1.91) | 3.02 (2.02-4.50) | <0.001 |  |
| Emphysema |  | | | | |  | | | | |  | | | | |
| Yes | 1.00 (Reference) | 1.57 (1.15-2.16) | 3.61 (1.38-9.45) | <0.001 | 0.724 | 1.00 (Reference) | 1.48 (0.83-2.65) | 2.27 (0.28-18.41) | 0.135 | 0.537 | 1.00 (Reference) | 1.64 (1.13-2.40) | 4.39 (1.48-13.08) | <0.001 | 0.382 |
| No | 1.00 (Reference) | 1.66 (1.47-1.87) | 2.70 (1.93-3.76) | <0.001 |  | 1.00 (Reference) | 1.73 (1.41-2.13) | 2.29 (1.24-4.23) | <0.001 |  | 1.00 (Reference) | 1.62 (1.40-1.89) | 2.91 (1.96-4.33) | <0.001 |  |
| Chronic bronchitis |  | | | | |  | | | | |  | | | | |
| Yes | 1.00 (Reference) | 1.50 (1.09-2.07) | 1.90 (1.02-3.54) | 0.003 | 0.357 | 1.00 (Reference) | 1.38 (0.79-2.42) | 0.75 (0.18-3.17) | 0.608 | 0.038 | 1.00 (Reference) | 1.60 (1.08-2.36) | 2.84 (1.40-5.75) | <0.001 | 0.634 |
| No | 1.00 (Reference) | 1.68 (1.49-1.90) | 3.15 (2.19-4.53) | <0.001 |  | 1.00 (Reference) | 1.77 (1.44-2.19) | 3.21 (1.70-6.08) | <0.001 |  | 1.00 (Reference) | 1.64 (1.41-1.90) | 3.12 (2.00-4.85) | <0.001 |  |
| Arthritis |  | | | | |  | | | | |  | | | | |
| Yes | 1.00 (Reference) | 1.46 (1.26-1.69) | 2.31 (1.61-3.32) | <0.001 | 0.001 | 1.00 (Reference) | 1.51 （1.17-1.95） | 1.39 （0.65-2.99） | 0.003 | 0.015 | 1.00 (Reference) | 1.43 (1.19-1.72) | 2.83 (1.87-4.28) | <0.001 | 0.022 |
| No | 1.00 (Reference) | 2.02 （1.70-2.40） | 4.76 （2.57-8.79） | <0.001 |  | 1.00 (Reference) | 2.02（1.50-2.73） | 5.78（2.30-14.54） | <0.001 |  | 1.00 (Reference) | 2.03 (1.64-2.50) | 4.13 (1.81-9.43) | <0.001 |  |
| Cancer |  | | | | |  | | | | |  | | | | |
| Yes | 1.00 (Reference) | 1.51 (1.18-1.93) | 2.15 (1.23-3.75) | <0.001 | 0.101 | 1.00 (Reference) | 1.57 (1.00-2.45) | 2.07 (0.81-5.31) | 0.017 | 0.475 | 1.00 (Reference) | 1.49 (1.11-2.00) | 2.19 (1.10-4.39) | <0.001 | 0.127 |
| No | 1.00 (Reference) | 1.67 (1.47-1.90) | 3.57 (2.45-5.21) | <0.001 |  | 1.00 (Reference) | 1.68 (1.35-2.09) | 2.33 (1.09-4.97) | <0.001 |  | 1.00 (Reference) | 1.67 (1.43-1.96) | 4.29 (2.77-6.64) | <0.001 |  |
| Liver dysfunction |  | | | | |  | | | | |  | | | | |
| Yes | 1.00 (Reference) | 1.54 (0.91-2.61) | 22.11 (6.41-76.28) | 0.001 | 0.006 | 1.00 (Reference) | 1.51 (0.62-3.71) | - | 0.435 | 0.038 | 1.00 (Reference) | 1.53 (0.79-2.97) | 50.93 (12.80-202.63) | <0.001 | 0.056 |
| No | 1.00 (Reference) | 1.62 (1.44-1.82) | 2.50 (1.81-3.47) | <0.001 |  | 1.00 (Reference) | 1.62 (1.32-1.98) | 2.20 (1.22-3.95) | <0.001 |  | 1.00 (Reference) | 1.62 (1.41-1.87) | 2.66 (1.80-3.94) | <0.001 |  |
| Moderate or severe nephropathy |  | | | | |  | | | | |  | | | | |
| Yes | 1.00 (Reference) | 1.70 (1.45-2.00) | 2.34 (1.56-3.50) | <0.001 | 0.344 | 1.00 (Reference) | 1.82 (1.40-2.38) | 2.11 (1.02-4.39) | <0.001 | 0.129 | 1.00 (Reference) | 1.64 (1.33-2.00) | 2.45 (1.51-3.97) | <0.001 | 0.906 |
| No | 1.00 (Reference) | 1.58 (1.34-1.85) | 3.15 (1.91-5.21) | <0.001 |  | 1.00 (Reference) | 1.45 (1.09-1.93) | 2.30 (0.85-6.25) | 0.003 |  | 1.00 (Reference) | 1.64 (1.35-1.98) | 3.62 (2.02-6.47) | <0.001 |  |
| Follow-up periods |  | | | | |  | | | | |  | | | | |
| ≤ 10 years | 1.00 (Reference) | 1.41 (1.24-1.59) | 2.66 (1.91-3.70) | <0.001 | 0.047 | 1.00 (Reference) | 1.53 (1.24-1.89) | 2.01 (1.09-3.71) | <0.001 | 0.018 | 1.00 (Reference) | 1.35 (1.16-1.58) | 3.04 (2.05-4.51) | <0.001 | 0.451 |
| > 10 years | 1.00 (Reference) | 1.26 (0.96-1.64) | 1.75 (0.70-4.36) | 0.047 |  | 1.00 (Reference) | 0.95 (0.56-1.56) | 1.19 (0.16-8.85) | 0.867 |  | 1.00 (Reference) | 1.43 (1.05-1.95) | 2.03 (0.73-5.65) | 0.011 |  |

S4 Table. Subgroups analyses of the effect of GPS level on adverse events based on Model 3. Model 3 is adjusted for age, sex, race, education status, smoking, poverty-income ratio, body mass index, triglyceride, total cholesterol, diabetes mellitus, hypertension, asthma, chronic bronchitis, arthritis, cancer, emphysema, liver dysfunction, and moderate or severe nephropathy. CI: confidence interval, GPS: Glasgow Prognostic Score, HR: hazard ratio.

## S5 Table. Cox regression analysis for the post hoc analysis outcomes

| **S5 Table. Cox regression analysis for the post hoc analysis outcomes** | | | | | | | | | | | | | | | | | | | | |
| --- | --- | --- | --- | --- | --- | --- | --- | --- | --- | --- | --- | --- | --- | --- | --- | --- | --- | --- | --- | --- |
| Outcomes | PLR, HR (95% Cl) | | | | NLR, HR (95% Cl) | | | | Total white blood cell count, HR (95% Cl) | | | | CRP, HR (95% Cl) | | | | Albumin, HR (95% Cl) | | | |
|  | 0 | 1 | 2 | P for trend | 0 | 1 | 2 | P for trend | 0 | 1 | 2 | P for trend | 0 | 1 | 2 | P for trend | 0 | 1 | 2 | P for trend |
| All-cause death |  |  |  |  |  |  |  |  |  |  |  |  |  |  |  |  |  |  |  |  |
| Model 3 | 1.00 (Reference) | 1.12  (1.01-1.24) | 1.05 (0.95-1.16) | 0.366 | 1.00 (Reference) | 1.22 (1.10-1.36) | 1.49 (1.35-1.66) | <0.001 | 1.00 (Reference) | 1.13 (1.02-1.24) | 1.44 (1.30-1.59) | <0.001 | 1.00 (Reference) | 1.09 (0.98-1.21) | 2.08 (1.88-2.31) | <0.001 | 1.00 (Reference) | 1.25 (1.13-1.38) | 1.86 (1.67-2.08) | <0.001 |
| P Value |  | 0.029 | 0.331 |  |  | <0.001 | <0.001 |  |  | 0.021 | <0.001 |  |  | 0.119 | <0.001 |  |  | <0.001 | <0.001 |  |
| Cardiac death |  |  |  |  |  |  |  |  |  |  |  |  |  |  |  |  |  |  |  |  |
| Model 3 | 1.00  (Reference) | 1.20 (1.01-1.43) | 1.11 (0.93-1.33) | 0.262 | 1.00  (Reference) | 1.29 (1.07-1.55) | 1.64 (1.36-1.96) | <0.001 | 1.00 (Reference) | 1.12 (0.94-1.32) | 1.29 (1.08-1.54) | 0.005 | 1.00 (Reference) | 1.14 (0.95-1.36) | 2.11 (1.76-2.52) | <0.001 | 1.00 (Reference) | 1.16 (0.97-1.37) | 1.74 (1.43-2.10) | <0.001 |
| P Value |  | 0.036 | 0.232 |  |  | 0.007 | <0.001 |  |  | 0.205 | 0.005 |  |  | 0.161 | <0.001 |  |  | 0.098 | <0.001 |  |
| Non-cardiac death |  |  |  |  |  |  |  |  |  |  |  |  |  |  |  |  |  |  |  |  |
| Model 3 | 1.00 (Reference) | 1.08 (0.95-1.22) | 1.02 (0.91-1.1*6*) | 0.739 | 1.00 (Reference) | 1.19 (1.05-1.36) | 1.43 (1.26-1.62) | <0.001 | 1.00 (Reference) | 1.13 (1.00-1.28) | 1.51 (1.34-1.72) | <0.001 | 1.00 (Reference) | 1.06 (0.93-1.21) | 2.07 (1.82-2.35) | <0.001 | 1.00 (Reference) | 1.30 (1.14-1.47) | 1.93 (1.68-2.22) | <0.001 |
| P Value |  | 0.231 | 0.711 |  |  | 0.007 | <0.001 |  |  | 0.055 | <0.001 |  |  | 0.366 | <0.001 |  |  | <0.001 | <0.001 |  |

S5 Table. Cox regression analysis for the post hoc analysis outcomes. Model 3 is adjusted for age, gener, race, education status, smoking, poverty-income ratio, body mass index, triglyceride, total cholesterol, diabetes, hypertension, asthma, emphysema, chronic bronchitis, arthritis, cancer, liver dysfunction, and moderate or severe nephropathy. CI: confidence interval, CRP: C-reactive protein, confidence interval, NLR: neutrophil-to-lymphocyte ratio, PLR: platelet-to-lymphocyte ratio, HR: hazard ratio.

## S4 Fig. Consistent Association: GPS with Mortality Risk in Cardiovascular Disease Patients


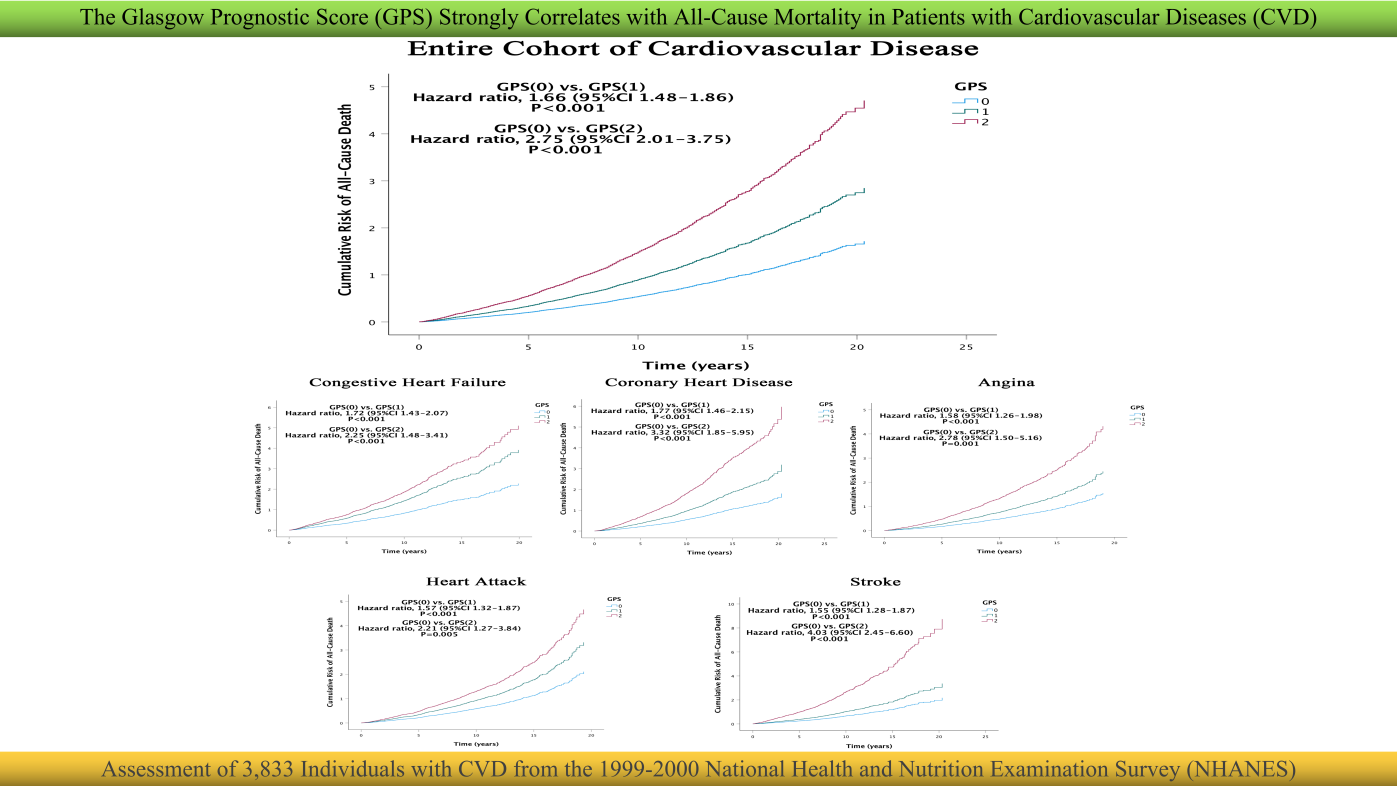


S4 Fig. Consistent Association: GPS with Mortality Risk in Cardiovascular Disease Patients. CI: confidence interval, GPS: Glasgow Prognostic Score.
